# Barriers and facilitators to integrating depression care in tuberculosis services in South Asia: a multi-country qualitative study

**DOI:** 10.1186/s12913-023-09783-z

**Published:** 2023-07-31

**Authors:** Olamide Todowede, Saima Afaq, Anoshmita Adhikary, Sushama Kanan, Vidhya Shree, Hannah Maria Jennings, Mehreen Riaz Faisal, Zara Nisar, Ikram Khan, Geetha Desai, Rumana Huque, Najma Siddiqi

**Affiliations:** 1 University of York, York, England, UK; 2 University of Nottingham, Nottingham, England, UK; 3 School of Public Health, Faculty of Medicine, Imperial College London, London, England, UK; 4 Khyber Medical University, Peshawar, Pakistan; 5 National Institute of Mental Health and Neurosciences, Bengaluru, Karnataka India; 6 ARK Foundation, Dhaka, Bangladesh; 7 Hull York Medical School, York, England, UK; 8 Bradford District Care NHS Foundation Trust, Bradford, England, UK

**Keywords:** Depression, Tuberculosis, India, Bangladesh, Pakistan, LMIC, Integrated care

## Abstract

**Background:**

Depression is common among people with tuberculosis (TB). The condition is typically unrecognised or untreated despite available and effective treatments in most low- and middle-income countries. TB services in these countries are relatively well established, offering a potential opportunity to deliver integrated depression screening and care. However, there is limited evidence on how such integration could be achieved. This study aimed to understand the barriers and facilitators to integrate depression care in TB services.

**Methods:**

We conducted nine workshops with 76 study participants, including people with TB, their carers, and health service providers in Bangladesh, India, and Pakistan, seeking views on integrating depression care into TB clinics. We used a deductive thematic approach to analyse the translated transcripts of audio recordings, contemporaneous notes made during workshops for Bangladesh and India and workshop reports for Pakistan. Using the SURE (Supporting the Use of Research Evidence) thematic framework, we extracted and categorised barriers and facilitators into various domains.

**Results:**

Reported barriers to integrating depression care in TB services included lack of knowledge about depression amongst patients and the staff, financial burden, and associated stigma for people with TB and their carers. Government buy-in and understanding of how to identify and screen for depression screening were potential facilitators reported. Additionally, breaking through mental health stigma and providing the additional resources required to deliver this service (human resources and consultation time) were essential for integrating depression and TB care.

**Conclusions:**

Depression is a common condition found among people with TB, requiring early **i**dentification among people with TB. Integrating depression care into Tb services by health workers requires the availability of political support and the provision of resources.

**Supplementary Information:**

The online version contains supplementary material available at 10.1186/s12913-023-09783-z.

## Background

Tuberculosis (TB) is a significant contributor to the global burden of diseases, especially in low- and middle-income countries (LMICs) [1]. The most significant burden of TB is in South Asia(44%); Bangladesh (3.6%), India (26%) and Pakistan (5.7%) are among the eight countries that contribute two-thirds of the total number of TB cases in the world [2]. The Sustainable Development Goal (SDG) target 3.3 requires an 80% reduction in TB incidence by 2030; the World Health Organisation has approved a target to achieve a 90% reduction in TB incidence and a 95% reduction in TB mortality by 2035 [2, 3]. Although the adoption of directly-observed therapy short-course (DOTS) has been successful in improving tuberculosis outcomes [4–7], nevertheless these ambitious targets cannot be achieved unless comorbid conditions, which commonly occur alongside TB, and adversely affect its outcomes, including mortality, are managed adequately [4–6].

Mental health problems are highly prevalent in people with tuberculosis. They may affect TB treatment adherence, leading to poor health outcomes such as treatment failure, multidrug-resistant TB, increased mortality and healthcare expenditure [8]. Depression is the most common mental health condition in people with TB [9–11]. The two conditions share a complex and bidirectional interaction, each being a risk factor for the development of the other [12, 13]. A meta-review reported a global pooled depression prevalence of up to 45% (95% CI: 38.04–52.55%) in people with TB [14]. The prevalence of depression in people with TB was higher than those without TB in a cross-sectional data analysis across 48 low and middle-income countries (23.7% vs. 6.8%, respectively) [7]. A higher rate of depression among the TB population has been reported in Bangladesh (33.8%) [15], India (23.6%) [16] and Pakistan (42.8%) [17].

Tuberculosis treatment services are relatively well established and embedded within most primary, secondary, and tertiary care settings in these LMICs. Conversely, depression is treatable with relatively simple, low-cost psychological or pharmacological therapies. Still, healthcare services usually fail to diagnose or treat it in LMIC settings, particularly in people with TB [18]. Preliminary evidence supports integrating mental health care (counselling and psychological interventions) into TB care [18–20]. This could potentially lead to better detection and treatment of depression and increase the quality of care and quality of life for TB patients, reduce healthcare costs, provide more patient-centred care and improve TB treatment completion, and ultimately save lives [18, 21, 22]. Embedding depression screening and treatment using a simple, scalable intervention in TB care offers an opportunity to improve the care of depression and TB outcomes. It can also help address the well-recognised mental health treatment gap, as many people with mental disorders do not have access to evidence-based treatments globally, especially in LMIC. A lack of mental health specialists in LMICs contributes to the large ‘treatment gap’ for mental disorders [23].

There is general recognition of the strong associations and inter-related causal mechanisms between mental disorders and chronic physical health problems, necessitating a joint approach to care [24]. WHO’s mental health action plan 2013-20 calls for “the integration of mental health treatment into general hospitals and primary care” [25]. United Nations member states recently agreed to expand their focus on the big four NCDs, including the fifth, mental disorders and argued for a joint approach [26]. The process of embedding screening and care for depression in TB care aligns with this agenda. Task shifting (non-specialist health or lay workers trained and supervised to deliver interventions usually provided by specialists) [27] can help this shortage and is supported by the WHO mhGAP [28] as a way to bridge current unacceptable treatment gaps. Moreover, healthcare systems have historically separated services for mental and physical health.

Despite the potential benefits of integrating care for depression in TB services, little is known about how it can be achieved [9, 29]. To address this gap, the present study aimed to understand the facilitators and barriers to detecting and treating depression in people with TB from the perspective of diverse TB stakeholders, including patients, their families and caregivers, healthcare professionals and policymakers in Bangladesh, India and Pakistan.

## Methods

### Study design and participants

This study is part of a research programme to design and evaluate an integrated depression care pathway in TB services, following the MRC framework for complex interventions [30–33]. This study reports the development phase of our research programme, allowing stakeholders to create realistic expectations, impacts and outcomes within their country’s context and available resources [34]. This study focuses on understanding the barriers and facilitators to deliver an integrated pathway for screening and care of depression in Bangladesh, India and Pakistan, considering the uncertainty of the development and acceptability of a culturally appropriate pathway for case finding and treatment of depression, integrated with TB services in these settings.

We conducted a series of iterative workshops to elicit views on the barriers, facilitators, existing gaps and opportunities for integrating depression care in TB services. We engaged people with TB (or recovered patients), families, health professionals (mental health experts, TB/chest medicine experts and DOTS facilitators), public health experts and policymakers. These study participants were identified through the study team’s existing clinical and research networks in each country, ensuring the representation of service providers and users.

### Data collection

All participants were given a study description, and written informed consent was obtained. Workshops were conducted as hybrid physical and online meetings with a mix of participants. Workshops were performed separately for health professionals and people with TB infection to manage the power dynamics between the two groups. A workshop guide was developed for facilitators to outline the format and included questions and prompts that could be used to explore facilitators and barriers to integrated care (Appendix 1). Workshops consisted of a presentation, in lay language, giving an overview of depression and TB (definitions of depression and TB, information on the prevalence of depression among TB patients and the impact and current knowledge of depression-TB care management) and the aim and rationale for developing depression case-finding and care pathways. After the presentation, participants discussed their experiences and perspectives on depression and TB and factors that could facilitate or hinder the implementation of integrated depression-TB screening and treatment in their setting.

Workshop facilitators were explicitly trained to present and share information, ask open-ended questions, probe, engage, and ensure mutually respectful, inclusive participation, allowing everyone to give their views. The workshops were conducted in Bangla (in Bangladesh), Urdu (in Pakistan), and a mixture of Kannada, English and Hindi (in India).

### Data management and analysis

Each workshop was recorded, transcribed verbatim and translated into English by researchers in Bangladesh (SK) and India (AA & VS), while in Pakistan, contemporaneous notes were made, from which a detailed report was prepared (in English) of the workshops (SA). Analysis was conducted using a deductive thematic approach. OT and AA analysed the data following the SURE (Supporting the Use of Research Evidence) thematic framework [35]. The SURE framework assists in improving decision-making about health systems in sub-Saharan Africa by enhancing policymakers’ access to and use of evidence-based research [36]. The framework is divided according to domains related to *providers of care, recipients of care, other related stakeholders, health system constraints* and *social and political constraints* (Appendix 2). This framework has been increasingly used in a low-resource setting, Khumalo et al. 2020 used it to understand the barriers and facilitators of rendering HIV services by community health workers in sub-Sahara Africa [37], and Musinguzi et al. 2022 categorising the barriers and facilitators that might affect providers of care to utilise Uganda Ministry of Health framework [38]. The framework has been used to assess factors affecting task-shifting implementation in midwifery service [39]. Therefore, we analysed our results using this framework to assess barriers and facilitators that could impact healthcare providers and recipients of care during the integrated pathway for depression screening and care in TB services.

OT and AA read through all the workshop transcripts and reports and met to discuss, reflect, and compare notes. They devised a coding framework based on the SURE domains and extracted barriers and facilitators from the transcripts. Findings were initially analysed and synthesised separately for each country and then compared across countries, examining cultural differences across the three countries.

### Ethical considerations

The study was approved by the Health Sciences Research Governance Committee (***HSRGC/2020/418/D)*** of the University of York. Ethical approval was also received from the Institute of Health Economics (IHE) in Bangladesh, the National Institute of Mental Health and Neurosciences (NIMHANS) in India and KMU **(Dir/KMU-EB/IPHSS/MM-001**) in Pakistan. Written informed consent was received from all study participants.

## Results

### Demographic characteristics

A total of 76 study participants were involved in a series of 9 workshops across Bangladesh (N = 4), India (N = 3) and Pakistan (N = 2) between February and April 2021. Study participants included (i) individuals who were receiving or had completed TB treatment and their carers (N = 21), (ii) Specialised clinicians including chest medicine experts and mental health experts (N = 16), (iii) TB control program staff including DOTS facilitators, medical officers, and managers (N = 19) and (iv) policymakers N = 3). The study participants’ ages ranged from 17 to 65 years, and 35 were women - Table 1.

**Table 1:** Characteristics of 76 workshop participants across the three countries

Characteristics	Bangladesh (n = 19) (n/N=%)	India (n = 30)(n/N=%)	Pakistan (n = 27) (n/N=%)
**Sex**			
Male	7(36.8%)	12(40%)	22(81.5%)
Female	12(63.2%)	18(60%)	5(18.5%)
**Health care providers**	6(31.6%)	17(56.7%)	15(55.6%)
Clinicians & Specialist	-	5(16.7%)	7(25.9%)
TB Project Management	3(15.8%)	1(3.3%)	4(14.8%)
TB-DOTS Program Staff	3(15.8%)	10(33.3%)	1(3.7%)
Policy Maker	-	1(3.3%)	3(11.1%)
**People with TB**	8(42.1%)	9(30.0%)	6(22.2%)
**TB Carers**	5(26.3%)	4(13.3%)	6(22.2%)

### Overview of findings

The barriers and facilitators to integrating depression care into tuberculosis services reported by workshop participants are summarised in Table 2, organised by relevance to (1) recipients of care, (2) providers of care and (3) health systems constraints, and by SURE Framework concepts.

**Table 2:** Summary of Barriers and Facilitators to Integration across various levels

Level	SURE Framework concepts	Barriers	Facilitators
Recipients of care (TB patients and carers)	Knowledge and skills	● Lacks knowledge about their mental health issues and relatedness to TB with TB diagnosis and treatment. ● Lack of clear communication with health professionals. ● Patients are unaware of their mental health and how well to get diagnosed, treat, and manage it.	● Knowledge of depression, stress, and anxiety symptoms because of their TB diagnosis and treatment. ● Realisation that their overall well-being (physical and mental) deteriorates due to TB stigma, treatment interaction and social isolation. ● Awareness that mental health workers can assist in managing and treating their depression symptoms. ● Carers’ awareness of the changes in the mental health of TB patients after diagnosis and during treatment and willingness to assist patients in coping with their symptoms and care.
	Attitudes regarding programme acceptability, appropriateness, and credibility	● Lack of diagnosis confirmation when TB patients experience depression symptoms. ● Patients with TB and caregivers were worried about the additional medical care costs should depression care be included in their ongoing services. ● Limited consultation time between health workers and patients. ● Patients are only willing to spend limited time at the hospital because of their TB fragility. ● Patients are dissuaded from discussing their depression symptoms because of health workers’ attitudes and unfriendliness. ● Unwillingness to discuss mental well-being and depression symptoms with health workers of the opposite gender. ● Poor perception that public hospital health workers do not pay quality attention and provide patients guidance. ● Preference for private healthcare practice over public government hospitals. ● High community TB stigma prevents patients from speaking about their mental health.	● Perceived competence of healthcare staff to care for their mental health. ● Enthusiastic about taking care of both their physical and mental health. ● Perceived willingness to accept depression care if provided with their TB treatment programme. ● Agreement that integrated depression services into current TB care will be efficient and economical, helpful and acceptable. ● Willingness to be screened for depression and receive counselling for depression management, not medications.
	Motivation to change or adopt a new behaviour	● Lack of motivation to seek help when presented with depression symptoms due to the burden of patients’ multimorbidity conditions. ● Patients’ preferences for specific health workers. ● Limited mental health awareness and counselling from the health workers. ● Due to a reoccurring lack of trust and stigma between patients and healthcare workers, people with TB were worried that service providers might not keep their mental health diagnosis confidential.	● Acknowledgement of the importance and the need for depression screening and treatment with their TB treatment programme ● Availability of psychosocial support from carers. ● Ability to develop a coping strategy and support system for managing depression symptoms. ● Willingness of people with TB and their carers to be screened for depression, receive mental health counselling, should this be offered during the TB treatment programme.
*Provider of care*	Knowledge and skills	● Lack of knowledge on how to inquire and counsel TB patients about depression. ● Lack of training and confidence to identify, diagnose and treat depression. ● Lack of knowledge regarding depression screening tools and guidelines for TB patients. ● Belief that screening for depression was the role of mental health workers.	● Prior TB treatment counselling training. ● Acknowledgement that depression symptoms presentation is joint among people with TB and affects their treatment adherence. ● Health workers capable of holistically assessing patients. ● Knowledgeable about common risk factors such as alcohol and drug use among TB patients and its association with depression.
	Attitudes regarding programme acceptability, appropriateness, and credibility	● Overburdened with high patient inflow. ● Limited consultation time and space. ● Lack of guidelines on screening and managing depression in the TB care pathway. ● Lack of confidence and capacity to deliver quality depression screening and support. ● Perception that patients’ reluctance to discuss their mental health would affect the programme’s acceptability.	● Acknowledgement that depression symptoms are common comorbidity among TB patients requires urgency. ● Willingness to deliver depression screening and low-intensity support. ● Policymakers were willing to utilise existing resources to deliver depression screening, staff training and low-cost treatment plan.
	Motivation to change or adopt a new behaviour	● Perception that patients would reject counselling when offered by the health workers due to lack of trust. ● Belief that patients would be unwilling to talk about their mental health for fear of the stigma associated with mental illness. ● Increase in workload with the addition of depression screening as part of their work duties.	● Belief that screening for depression and counselling should be conducted from the onset of TB diagnosis. ● Willingness to have a TB clinic staff screen or treat depression if trained. ● Willingness to leverage the resources of established mental health care and facilities, for instance, launching mental health programme for the HIV-infected population ● Support for the idea of creating awareness about depression among tuberculosis patients.
*Health system constraints*	Resources	● The health system is under-resourced, with limited human resources in the tuberculosis clinics. ● Need to incentivise staff to engage in depression screening and referral process	● Acknowledgement that human resources can be managed and utilised for the screening. ● Budget allocation for recruitment of additional staff
	Management and leadership	● Lack of quality and affordable services may impact the integration of new programmes	● Buy-in from health ministries can facilitate integrating depression into services in the TB care pathway.

Findings are discussed below, grouped by (i) barriers and facilitators relevant to providers and recipients, (ii) those related to health systems and (iii) recommendations to support better-integrated care.

### Provider and recipient level barriers and facilitators

#### Knowledge and skills

Lack of knowledge, training, and capacity to identify and deliver depression care to patients was a predominant barrier mentioned by healthcare workers in all three countries. Healthcare providers discussed that current TB clinical practices do not include inquiries about mental health (including depression) from people with TB in their settings. In India and Pakistan, participants mentioned that people with tuberculosis are not well-sensitised about the symptoms of depression, and this is due to a lack of knowledge from health workers. Providers and recipients of care and recipient of care (people with TB and caregivers) acknowledged that a major significant challenge is the lack of guidelines and screening tools for depression screening among people with TB in their settings. In India, health workers stated that they lack the knowledge that depression care, such as screening and treatment, can be delivered by a non-mental health person where appropriate.

People with tuberculosis lacked knowledge about the increased likelihood of mental health problems for those with TB and were unaware of the availability of depression services. The lack of counselling about mental health issues from health workers during the delivery of tuberculosis services was stated by participants from all three countries.

*“I have heard that health workers for mental health are psychologists. As far as I’ve heard from them, they study this subject and then provide treatment; they will be the best people to treat depression” TB Patient, Bangladesh.*

*“Also, I’m not very confident about treating a psychiatric illness. at least, I may not be so confident to treat.” TB Consultant, Pakistan.*

This study found out that few of the TB care providers had some (but limited) (a) knowledge of depression symptoms, (b) prior training in counselling patients with TB, (c) understanding that people with TB require mental health support, (such as depression care); these potential facilitators can help deliver holistic care to patients with TB and depression. However, it was highlighted that TB healthcare workers often associate depression-related signs with TB rather than mental health itself. Hence, further sensitisation about mental health amongst TB care providers is essential to providing depression care to TB patients.

*“I think there’s a lot of misleading in symptoms because most of the patients do not come to us directly with symptoms of depression, and when they present to us with general symptoms, a loss of appetite or loss of weight, we obviously will attribute it to the disease process itself instead of thinking of the mental health” -Clinician Consultant, India.*

*“There is no healthcare education, and the staff cannot do counselling sessions due to lack of training. Capacity development should be done, and lower staff should be trained to diagnose and treat fundamental psychiatric problems in TB Patients: TB Program Manager, Pakistan.*

Across all the countries, the recipients of care acknowledged (a) that they have experienced or are still experiencing symptoms of depression, (b) are aware that their overall well-being deteriorates because of TB infection, stigma, and their state of emotional well-being, (c) the carers and patients are reliant on self and communal coping strategies for depression symptoms management, and (d) Some of the patients have had depression prescriptions in the past by the health workers.

#### Attitudes regarding integration acceptability, appropriateness, and credibility

Healthcare workers across all three countries reported being unsure of their capacity to deliver depression services. Health workers said they were overburdened with the number of people with tuberculosis that needed to be dealt with in their facilities (high patent flow) within limited space and time. The lack of integrated depression care guidelines into tuberculosis services is a significant barrier to implementation. None of the countries had a care plan or procedures for managing depression in pulmonary tuberculosis. Health workers spoke about patients’ reluctance to discuss their mental health. Some health workers felt that stigmatisation of mental health conditions such as depression might impact the acceptability of depression screening among patients.

The barriers related to acceptability, appropriateness, and credibility of integrating depression care expressed by people with TB were regarding their unwillingness to discuss their mental health issues with health workers because of confidentiality issues and lack of empathy by the health workers, the poor quality and short consultation time they received, and the lack of engagement by the health workers. In India, people with TB were concerned and unwilling to discuss their mental health with the opposite gender. For instance, due to cultural views, a male TB patient was reluctant to discuss his mental health issues with a female health worker. The gender difference was a significant barrier in discussing mental health-related issues between health workers, people with TB, and their caregivers. Thus, it might be a challenge for health workers to identify depression in their patients without the opportunity to start a conversation.

Furthermore, people with TB indicated that health workers pay little or no attention to them in government-funded facilities. They are not provided with comprehensive guidance about their health issues and their management, so they prefer using private healthcare facilities where they receive more focused attention and care. People with TB and their caregivers were concerned about the additional financial and medical costs associated with depression services.

*“I also had TB, then the doctor said from a distance, “Stay there, talk from there”, this is upsetting for someone with tuberculosis.” Caregiver, Bangladesh*.

*“Not only me but all TB patients will also welcome this idea. I would love to participate in this screening” TB patient, Pakistan.*

Healthcare providers across all countries mentioned (a) that depression is a common comorbidity among TB patients and requires urgent interventions, (b) healthcare workers were willing to deliver depression screening and low-intensity treatment, should they receive training, (c) policymakers were willing to utilise existing resources to deliver depression screening, staff training and low-cost treatment plan. For instance, health workers that were interviewed in India stated that delivering culturally appropriate depression awareness programs that involve TB patients and community members will encourage the adoption and acceptance of integrated depression-tuberculosis care within their context.

*“So as a clinician, I’ll be concentrating more on the tuberculosis part, and the clinical symptoms, the physical symptoms, and mental health is always something which is often neglected” Clinician, India.*

*“We found depression in maximum TB patients. They are mostly paranoid about whether or not they will be cured. A lot of depression and fear works inside them. Most of them are extremely concerned and scared.” DOTS facilitator, Bangladesh*.

People with TB and their carers acknowledged (a) the importance and benefits of depression screening alongside their TB treatment regime, (b) their willingness to be screened for depression and provided with quality counselling with more details about their mental health, (c) across all countries, the integration of depression care into TB services would be acceptable to the people with TB and their carers. In Bangladesh, the study participants emphasised that the availability of this care will be more appropriate than their current coping strategy of discussing with friends and families when they experience depression symptoms.

*“If the patient wants to say something regarding their depression to their family, they don’t understand. It is important to understand how to console the patient. That’s why I think it will greatly benefit if the patient receives counselling with the TB treatment here.” TB patient, Bangladesh*.

*“To accept depression care along with TB care will be helpful, and we can undergo it. Because even now you are providing us information on this, and so we can understand all this and so it will be beneficial” Carer, India*.

*“Yes, because it will be helpful to take care of both physical and mental health”, Carer, India*.

*“I will be ready to take it because it will help in better understanding the problem I am going through and also to overcome the same”, Carer, India.*

#### Motivation to change

Most of the health workers were not motivated to integrate depression care into TB care, as they had the perception that should depression care be offered, there would be a lack of engagement by the patients for depression screening and counselling due to the stigma associated with mental illness. Also, health workers in Bangladesh reported that depression screening would add to their workload. All healthcare workers and policymakers were uncertain about the referral guidance and process to follow should TB patients screen positive for depression. There was also a presumption of patients not accepting depression screening and treatment because of the patient’s lack of trust in the health care workers.

*“So if they (DOTS provider) are to provide counselling to the people, it would take up some extra time”, a Health worker (Manager) in Bangladesh.*

*“To understand the specific reason behind the depression and how to deal with it takes much time, and it would not be possible for the existing employee to manage the time for counselling barriers” Health worker, Bangladesh.*

*“Stigmatisation around TB does not allow patients to discuss or express themselves with the health workers; this might affect any form of discussion about mental health”. Clinician, Pakistan*.

Lack of rapport and relationship between health care providers and people with TB was a de-motivator for discussing their mental health issue with health care providers; culturally, such expression of sadness is considered to be mainly discussed with family and friends. People with TBs recalled that the lack of mutual respect from the health workers is a significant factor that could affect their ability to trust them with their mental health care.

Motivation to change-related facilitators mentioned by the providers of care includes the following: (a) the willingness to incorporate depression screening and treatment into current services by healthcare workers should there be training and additional resources, (b) the willingness to create more time for the provision of holistic care for TB patients beyond treatment, (c) acknowledgement that if the present staff were trained on depression identification, they would be able to deliver essential depression treatment alongside current practice. In India, healthcare workers and policymakers were open to discussing taking ownership of the TB-depression care integration process. In Pakistan, the policymakers were willing to leverage the mental health care resources (human and facilities) that are currently being used for people living with HIV, for people with TB. Healthcare workers and policymakers across the three-country study sites agreed that screening patients attending DOTs and TB clinics for depression is viable.

*“So if they are trained (referring to the grassroots level health workers), they’ll be trained with simple scoring tools, something that can help them to pick up the psychiatric illness, then I think that would be a better solution”, Psychiatric Consultant, Pakistan.*

“*They (TB Health visitors) are not physicians, and they can’t treat patients. Maybe if we have a one-month training program, they can be trained to do the counselling process. But not the pharmacotherapy is possible” Pulmonologist, India.*

*“They (TB Health Visitors) can be doing the basic counselling. Only identification and basic counselling and referral”. - Policy Maker, India*.

*“It would be good if depression care is integrated with the TB treatment service…But I think everyone needs to be trained before it is integrated. In terms of Human Resources, it might need a little extra human resource. What I mean is, I think we need someone who has a little specialisation in counselling. Or have studied counselling or are trained.” Health facility manager, Bangladesh*.

The following facilitators were mentioned by people with TB and their carers (a) they were in support and were willing to accept depression screening care if offered at the facility, (b) belief that the current health worker’s role (DOTS) for the TB patients, will assist in driving the uptake of depression care, (c) people with TB were curious to understand the reasons and how to handle depression symptoms and willing to seek help. All recipients of care indicated that integration of depression care would be a motivator for TB treatment adherence.

“*If we can talk to them (health workers), they’ll talk to us. Coming here (to the TB clinic) was a good suggestion, and we’ll feel comforted as we were able to come here and speak out”. TB patient, India*.

### Health systems-level barriers and facilitators

#### Resources (human and financial)

Lack of skills and the limited staff was key barrier expressed across the three countries. In India and Pakistan, policymakers were willing to leverage their limited resources and integrate depression care into their current services. The TB coordinators were reluctant to use their existing human resources to deliver depression care in Bangladesh. In Bangladesh, the TB coordinators were receptive to using other physical resources that are available such as office space and dedicating some more time to delivering depression care (although the time allocated to each patient will be dependent on the patient flow). In India, it was discussed that there is a budget allocation for recruiting a counsellor within the TB clinic, which can be used to recruit additional persons to conduct depression screening. In Pakistan, policymakers and health workers mentioned that the present human resources could be used in delivering depression care.

Furthermore, the health workers expressed the need for extra incentives, such as financial incentives for non-mental health staff screening and referring patients for depression care.

*“It (referring to depression identification) should be a brief assessment. If we incentivise to detect depression, it will probably be useful”, Senior Nodal Officer, India*.

*“To make this process sustainable and attainable, we will need to provide some monetary benefit to the Dots facilitators to carry out this with their existing work”, Senior Pulmonologist, Pakistan.*

#### Patient flow processes

Across all three countries, health workers are struggling with a high workload and many TB patients; thus, adding on depression screening would be an added burden stretching their already heavy workload. Health workers expressed that the referral pathway to mental health facilities, when required, should be efficiently designed without increasing the time spent in the hospital by people with TB.

In India, health workers stated that there is an ongoing mental health awareness campaign organised by non-governmental organisations in communities; this could assist in increasing the acceptability of integrated depression care. Health workers in India noted that there is a current referral pathway for suspected mental health stress. However, this isn’t specific to depression and often used for people with multi-resistant TB(MDR-TB), and nearby mental health facilities are well mapped out for efficient referral purposes.

#### Management and leadership

People with TB and caregivers indicated that additional programs, such as depression screening and treatment, could strain the current poor quality and affordable TB services they receive. Healthcare workers mentioned that no holistic TB care pathway or guidelines dedicated to mental health screening could be adopted for integrating TB depression care. Healthcare workers and policymakers emphasised the need to ensure the Indian Ministry of Health’s buy-in on the plan to integrate depression into TB services. TB coordinator in Bangladesh mentioned that the success and sustainability of integrated care depend on its adoption by the National TB programmes and the Ministry of Health, as TB programmes are funded through these parastatals.

*“This proposition should be led by the India Ministry of Health”*– TB Consultant, India.

### Recommendations to support better-integrated care

Recommendations on how to integrate depression screening into TB care and practices were discussed by people with TB, their caregivers and health workers in this study. There was broad agreement across study participants’ groups on some aspects of the screening uptake and delivery, but differences of opinion regarding other elements, e.g., who among the clinical staff should deliver screening and the means of financing the new role. The following practical recommendations were discussed and concluded.

### Staff training on depression finding and screening tool

Most participants favoured screening TB patients for depression but reported that screening for depression would be less acceptable to patients. A strong opinion was articulated across all participants that all health workers attending to TB patients should be required to know how to identify and screen for depression, as this is currently unavailable in the TB treatment programme. It was noted that because of the high number of patients that health workers had to attend, they would require a short depression screening tool, that is, easy to go through with the patients within a limited time of the patient’s consultation.

*“Existing staff should be tailor-trained in using short and simple tools for screening depression in TB patients. DOTS facilitators are available in every TB centre and should be utilised. Patients should be educated by running awareness campaigns involving local community leaders. Strong community relations need to be built for successful results’’ Policy Maker, Pakistan*.

*“In Tertiary Care hospital, if the IPD staffs treating TB patients are being sensitised on depressive symptoms identification, then definitely it will be another aspect to work out in identifying depression among patients suffering with TB”, Senior Nodal Officer, India.*

*“Health visitors are in a better position to be trained and also to teach them some basic counselling skills if they find something”, Senior Pulmonologist, India.*

### Development of integrated depression and TB pathway

There was a broad consensus across health workers’ groups that integrating depression into TB care was necessary. However, the current practice does not provide guidelines on how, when and which patients to screen for depression. They noted that a screening pathway and policy need to be developed as guidance for health workers. The guideline and pathway should stipulate the process of finding, treating, and referring TB patients for depression care.

*“In the program (referring to the National TB Elimination Program), to find out depressive symptoms, there are no such guidelines. TB-HVs are being taught to be counselled the patient”, Senior Program Manager from National TB Program, India*.

*“This integration will greatly help the patients, so we are willing to help as much as possible. Currently, no guidelines are available, but if we can develop a pathway to follow, that will be the first step in the right direction” Program Manager Provincial TB Control Program, Pakistan.*

*“When someone with TB comes to receive treatment and starts a medicine course, we offer counselling…Firstly, we tell them that taking medicine can have some side effects. And at the same time, we assure him that he will improve if he takes TB medicine… But there still has not been an individual treatment plan or guideline for such cases (Depression in TB patients).” TB Facility Manager, Bangladesh*.

### Measures for implementing the proposed screening

People with TB and their carers suggested that depression screening and counselling commence immediately after TB diagnosis. Improving the quality of TB care counselling offered by healthcare workers could be enhanced by introducing depression screening. It was also noted that a psychiatrist would be required to serve in a supervisory role to TB healthcare workers. Increased consultation time and quality of counselling about depression will improve the acceptability of such a novel and essential service. Health workers in India and Pakistan recommended increased depression sensitisation among TB patients.

*“They (TB Health Visitors) are not physicians; they can’t treat patients. Maybe if we have a one-month training program, they can be trained to do the counselling process. But not the pharmacotherapy is possible”, Pulmonologist, India.*

*“Dots Facilitators can do the screening provided they are trained first. As they are the first point of contact of the patient, a senior psychiatrist can supervise them, but I think they are fit for the role” of TB Control Program Manager, Pakistan.*

*“I only came to receive the medicines… But depression was not discussed. Because he is a doctor… and I thought he could help with my TB treatment, but it does not cover depression…We can talk to the health care worker (DOT provider) first … but it is important to check whether the provider is willing to provide the depression care services.” TB patient, Bangladesh*.

## Discussion

Our study generated new evidence regarding barriers and facilitators to integrating depression care within TB services in Bangladesh, India and Pakistan. Health workers with day-to-day contact with people with TB emphasised that identifying and treating depression is vital in providing appropriate and holistic care for people with TB. The barriers found are predominantly related to the stigma associated with depression and TB, cultural gender-based roles, poor interpersonal relationships between health workers and people with TB and the quality of healthcare workers’ counselling skills. These barriers might negatively impact the acceptability and possible integration of depression care into tuberculosis services. Furthermore, broader structural issues and human resource capacity for services were also perceived to influence the integration of depression care negatively.

Potential opportunities to facilitate the integration of depression care discussed include leveraging existing human resources, increasing awareness of mental health, improving linkage and referral services between mental health specialist care and TB services, government and developmental organisations’ buy-in, and increasing the capacity of health workers to reduce burnout. Establishing collaborative care models can help achieve integrated care, but organisational and financial issues reported in our study may affect its adoption within the health systems [40]. Our findings are consistent with a study from Brazil that reported potential barriers to integrating depression and TB treatment in primary care are strained and under-resourced health systems and recurring TB and mental health-related stigma. Moreover, providing mental health training and building trusted relationships between community health workers and people with TB could facilitate the adoption of integrated care [41]. Another study reported a lack of knowledge about workable mechanisms for inter-sectoral collaboration between mental health services and other health services among health workers and a lack of community and service user engagement in developing innovative, coordinated care, such as depression and TB care [42]. Similarly, it’s reported among Zambian populations that health workers had inadequate understanding and training on how to screen and treat mental health conditions in TB patients [43]. In Malawi, inadequate resources (human and financial resources), burnout and lack of intimate consultation rooms were key barriers to delivering integrated depression care [44]. Achieving successful implementation and adoption of integrated care requires buy-in from the government and investors to provide financial incentives to support the systems and health workers and adaptation across different settings to offer additional care.

Lack of capacity, resources and guidelines for screening and identifying depression among this population were reported in our study. Studies have shown that training tuberculosis healthcare workers, providing technical assistance and supervision by mental health specialists to retain the knowledge of identifying and screening depression increases the likelihood of successfully integrating depression care into tuberculosis services [30–32]. Some of our study participants were not motivated to take up the additional role of screening for depression among the TB population because of the high workload. Moreover, task shifting to provide mental health services may be harnessed to integrate these services; this could involve minimal services such as screening, counselling, and referrals [45]. Similarly, our study supports the patient’s and public perspective that a critical element to addressing mental health issues among TB patients is to provide mental health support from the point of TB diagnosis until people with TB have fully recovered [30].

The current study showed the analysis of the results collated across three South Asian countries, but we acknowledge that the health systems vary within the three countries. For instance, one of the facilitators emphasised among health workers and policymakers in Pakistan is the willingness of the health workers to leverage the established HIV mental health services for people with TB. This is an established concept within different countries and HIV facilities [46], though scarcely documented in the region of study. In our research, the provider of care’s poor counselling capacity and behaviour was stated as a common barrier in India; these factors have been reported to cause delays in the TB care cascade in the country [47]. Strengthening the systems might not be sufficient to address the complexity of integrated care, but addressing the need to build in counselling capacities of the TB care providers. The resistance to adopting integrated care by healthcare workers in Bangladesh was mainly because the TB facilities are majorly externally funded, such as the Global Fund, with rigid funding and implementation plan [48, 49]. In the future, we suggest government parastatals incorporate service users’ needs into the country’s strategic goals and health programmes.

### Strength and limitations

The strength of our study is that we used a participatory approach that considers the views of recipients of care (people with TB) to understand how to improve healthcare services in low and middle-income countries. This approach informs transformative implementation strategies, generating realistic and culturally appropriate research outcomes targeting end users’ needs [50]. We actively engaged with people with TB, carers, health workers, public health experts and policymakers working on TB programmes in South Asia. We conducted separate workshops for people with TB, their carers and other stakeholders such as policymakers and health workers to ensure a power balance and encourage those with tuberculosis to share their opinions without intimidation.

We conducted this study during the COVID-19 pandemic. Still, we ensured that all participants adhered to the country-specific guidance (limited number of participants per workshop, masks, and social distancing) when attending in-person workshops. We realised that extensive discussions were hampered due to social distancing and wearing masks. The online workshops, while they were more accessible to some participants, resulted in limited interactions between study participants and patients with limited access to technology were excluded. Though the study planned to include participants from various backgrounds, we could not reach out to some groups, such as TB non-governmental/community organisations, tuberculosis activists, and global fund representatives, due to the impact of COVID-19 and accessibility issues. Worthwhile to note that the Bangladesh facility is funded through developmental donor funding, which influenced the worker’s reluctance to take up new roles outside their usual contracts. In Pakistan, the group discussion was not recorded, and we relied on handwritten reports taken during the interviews.

## Conclusion

Our study found that TB service providers in the three countries of interest encounter many TB patients with depressive symptoms. However, successful integration of depression into tuberculosis clinics can be achieved should the following be improved: health workers counselling capacities to identify and screen for depression, increased human and facility resources, and government and other investors’ buy-in are addressed. Creating awareness about depression among people with TB and their carers from the inception of their TB treatment programme could reduce the dual stigma of TB and depression. Although our study did not capture all the complex factors that could affect the implementation of integrated care, we recommend that in achieving TB elimination by 2050, policymakers and other stakeholders address measures of efficiently implementing collaborative care, such as mental health and TB services.

## Electronic supplementary material

Below is the link to the electronic supplementary material.

Supplementary Material 1

## Data Availability

Due to the sensitivity of the information, the data will not be uploaded to a public repository. However, the data is available upon request to the corresponding author.
