# Supplementary material for: Barriers and facilitators to integrating depression care in tuberculosis services in South Asia: a multi-country qualitative study
Source: BMC Health Serv Res. 2023 Jul 31;23:818. doi: 10.1186/s12913-023-09783-z (PMC10391993; doi:10.1186/s12913-023-09783-z)
Supplement: Supplementary file 1 — Supplementary Material 1 [file 12913_2023_9783_MOESM1_ESM.docx]

**Barriers and facilitators to the integration of depression care in tuberculosis services in South Asia: a multi-country qualitative study.**

Appendix 1: Topic guide for Workshops with Stakeholders

TOPIC GUIDE FOR HEALTH WORKERS AND OTHER STAKEHOLDERS

"Good morning. I am (facilitator introduces him/herself), and I will be facilitating this stakeholder consultation workshop.

This workshop is being conducted to get your views about the barriers, facilitators, existing gaps and opportunities for integrating depression care in tuberculosis services in your settings. This study will inform our research that aimed at identifying and managing depression in tuberculosis.

As stated in the information sheet, I will be recording our conversation. The purpose of this is so that I can get all the details, and at the same time be able to carry on an attentive conversation with you. I assure you that all your comments will remain confidential and anonymised.

The facilitator confirms that all *participants have* read, understood the information sheet, consented and signed the consent form. If yes, the stakeholder workshop can commence.

Section 1:

1. I'd like to start by having you briefly describe your responsibilities and involvement with this facility, tuberculosis patients, tuberculosis care and management – Key actors and intermediate actors. (Note to facilitator: You may need to probe to gather the information on the stakeholders that are participating).

Section 2: The facilitator will give a brief presentation on the project (aims and objective, the country’s contextual background on tuberculosis and depression comorbidity)

Section 3: We will be discussing some points related to the aim of the study, and I will ask you to discuss to the best of your knowledge and experiences, and answer in an open-ended manner. Focus on your practice experiences with tuberculosis and depression comorbidity.

 (Please ensure that participants are comfortable and that they can express themselves, stakeholders can be split into groups to brainstorm and share their thoughts, they can be given materials to note their discussions, recommendations and strategies, participants can be given activities to map out facilities and resources around and group hem under barriers and facilitators, case study or scenarios can be presented by facilitators, and other cultural context activities and plans. Let the participants know that there is no idea that is not worth discussing, so feel free to talk as much as you can within our limited time.)

Suggested questions (Facilitators should note that these are suggestive questions to get participants talking on the topic).

1.      How often do you see TB patients presenting with symptoms of depression?

2.      Do you discuss with TB patients about their mental health?

3.      How important do you think for depression care to be integrated into TB care and management?

4.      Are their guidelines or a treatment plan for TB patients that have symptoms of depression, please explain, and specify if they are available.

5.  In your opinion how important do you think depression services are to TB patients and how do you think this can be incorporated into TB services?

6.  What are the resources (human, financial or asset) that are presently available and can facilitate the integration of depression care into your present TB management and care services?

7.  Are there any policies or guidelines that can facilitate the integration of depression care into TB services?

8.  What are the issues/barriers that might arise with the integration of depression care into TB services in your context?

9.  What are your suggestions on how the issues or barriers stated above can be surmounted?

If not mentioned, probe about who (which staff) could deliver screening and treatment of depression.

Section 4: The researchers will develop the recordings and output of section 4 and produce a report that will be presented to the stakeholders in another workshop, to reach a consensus. 

TOPIC GUIDE FOR PEOPLE WITH TUBERCULOSIS

Name of Facilitator:

Date and Time of Facilitation:

The venue of Workshop:

Format of the workshop: Online (name the mode) or Physical **contact**

"Good morning. I am (facilitator introduces him/herself), and I will be facilitating this stakeholder consultation workshop.

This workshop is being conducted to get your views about the acceptability of routine depression case-finding and treatment as part of TB care and potential adverse outcomes of integrating these services into your care or that of your relative (speaking to carers). This study will inform our research that aimed at identifying and managing depression in tuberculosis.

As stated in the information sheet, I will be recording our conversation. The purpose of this is so that I can get all the details, and at the same time be able to carry on an attentive conversation with you. I assure you that all your comments will remain confidential and anonymised at the point of translating our conversation.

The facilitator confirms that all *participants have* read, understood the information sheet, consented, and signed the consent form. If yes, the stakeholder workshop can commence.

Please ensure that participants are comfortable and that they can express themselves, stakeholders can be split into groups to brainstorm and share their thoughts, they can be given materials to note their discussions, recommendations and strategies, Let the participants know that there is no idea that is not worth discussing, so feel free to talk as much as you can within our limited time.

Section 1: Suggested questions/discussion **points**

1. The facilitator can briefly describe what TB-depression comorbidity implies and what are the expectations of the study from the workshop in a simple term. To give the patients and carers depth of what is being talked about. (This can be done by painting scenarios, a case study of TB-depression comorbidity)
2. The facilitator can ask the participants to freely discuss if they have been affected by depression episodes (based on his/her discussion above) or going through that phase now. (Note to facilitator: You may need to probe to gather the information on the stakeholders that are participating).
3. The participants can be asked what they have done about the feeling (probing if they have asked health professionals, discussed with anyone), this is to explore their understanding about the subject matter and how important it is to them to seek assistance.
4. How would you feel if depression care is integrated into your TB care, irrespective if you have experienced what I have earlier stated? Do you think it will be worthwhile?
5. What are the potential adverse outcomes that might arise in integrating depression case finding and care into your present TB management plan?

Thank you!

Appendix 2:**Definition of concepts**

1. ***Knowledge and skills***

This is the degree of knowledge that the providers of care and recipient of care have about depression, integrating depression care in tuberculosis services, and the level of skills of applying the knowledge by the provider and recipient of care.

1. ***Attitudes regarding integration acceptability and appropriateness and credibility***

These are the opinions of the providers of care and recipients of care about the healthcare issues and the integration of depression screening into tuberculosis care. Their views about the acceptability and appropriateness of integrating depression care, and the credibility of the provider of the depression care and the healthcare system.

***(c)Motivation to change***

This is the degree of motivation by the provider and recipient of care to adopt integrated depression services into tuberculosis services.

***(d) Incentives***

This is the opinion on structured reimbursement systems needed for patients, health workers or others to facilitate rather than hinder implementation of integrated depression services into tuberculosis services.

**(e) Resources(Human & financial)**

Opinions on whether an increased supply or distribution of health workers or additional resources may be required to implement integrated depression services into tuberculosis services.

**(f) Patient flow process**

Views on the adequate processes for outreach and receiving, referring and transferring patients with depression may be needed for the implementation of integrated depression services into tuberculosis services.

**Appendix 3: Consolidated criteria for reporting qualitative research (COREQ): a 32-item checklist for interviews and focus groups**

| **No. Item** | **Guide questions/description** | **Reported on Page #** |
| --- | --- | --- |
| **Domain 1: Research team and reﬂexivity** | | |
| ***Personal Characteristics*** | | |
| **1. Interviewer/facilitator** | **Which author/s conducted the inter view or focus group?** | Sushama Kanan for Bangladesh, Saima Afaq for Pakistan, Anoshmita Adhikary and Vidhya Shree for India. |
| **2. Credentials** | **What were the researcher’s credentials? E.g. PhD, MD** | Sushama Kanan (MSc), Saima Afaq (PhD), Anoshmita Adhikary(MSc) and Vidhya Shree(MSc). |
| **3. Occupation** | **What was their occupation at the time of the study?** | Research Fellow |
| **4. Gender** | **Was the researcher male or female?** | Female and Male |
| **5. Experience and training** | **What experience or training did the researcher have?** | All researchers involved have been trained in the conduct of research, with the minimum of two years’ experience of conducting research. The research team is managed and coordinated by a Professor of Psychiatry with more than 15 years experience. |
| ***Relationship with participants*** | | |
| **6. Relationship established** | **Was a relationship established prior to study commencement?** | Participants were contacted prior to the study commencement to inform about the study objectives and brief about their role for cooperation. The study had an established community panel prior to the study, that meets quarterly. |
| **7. Participant knowledge of the interviewer** | **What did the participants know about the researcher? e.g. personal goals, reasons for doing the research** | Participants were provided with an information sheet and consent form which outlined the aim of the study. |
| **8. Interviewer characteristics** | **What characteristics were reported about the inter viewer/facilitator? e.g. Bias, assumptions, reasons and interests in the research topic** | The selection of some of the participants was a convenient selection. Participants knew that researchers were interested in designing a study looking at integrating depression care into TB services. |

| **Domain 2: study design** | | |
| --- | --- | --- |
| ***Theoretical framework*** | | |
| **9. Methodological orientation and Theory** | **What methodological orientation was stated to underpin the study? e.g. grounded theory, discourse analysis, ethnography, phenomenology, content analysis** | Thematic Analysis |
| ***Participant selection*** | | |
| **10. Sampling** | **How were participants selected? e.g. purposive, convenience, consecutive, snowball** | Purposive sampling such as snowballig and convenience methods. |
| **11. Method of approach** | **How were participants approached? e.g. face-to-face, telephone, mail, email** | The study was conducted during the COVID pandemic, particpant were approached both face to face and via virtual system using zoom. |
| **12. Sample size** | **How many participants were in the study?** | 76 |
| **13. Non-participation** | **How many people refused to participate or dropped out? Reasons?** | None |
| ***Setting*** | | |
| **14. Setting of data collection** | **Where was the data collected? e.g. home, clinic, workplace** | Data was collected in an hybrid format, some were collected in a meeting room at the primary health care center and Zoom. |
| **15. Presence of non-participants** | **Was anyone else present besides the participants and researchers?** | No |
| **16. Description of sample** | **What are the important characteristics of the sample? e.g. demographic data, date** | Gender, Job role, country |
| ***Data collection*** | | |
| **17. Interview guide** | **Were questions, prompts, guides provided by the authors? Was it pilot tested?** | Yes there was a workshop guide and this was not piloted. The guide was read by all authors and agreed upon before being used for the meeting and workshop. |
| **18. Repeat interviews** | **Were repeat interviews carried out? If yes, how many?** | **No** |
| **19. Audio/visual recording** | **Did the research use audio or visual recording to collect the data?** | Data were audio recorded using zoom recording and a researcher took note some of the meetings. . |
| **20. Field notes** | **Were ﬁeld notes made during and/or after the inter view or focus group?** | No |
| **21. Duration** | **What was the duration of the inter views or focus group?** | Each meeting and workshops took a maximum of 60minutes |
| **22. Data saturation** | **Was data saturation discussed?** | Yes |
| **23. Transcripts returned** | **Were transcripts returned to participants for comment and/or correction?** | No |
| **Domain 3: analysis and ﬁndings** | | |
| ***Data analysis*** | | |
| **24. Number of data coders** | **How many data coders coded the data?** | **2** |
| **25. Description of the coding tree** | **Did authors provide a description of the coding tree?** | Yes ( Appendix 1, SURE framework coding sheet) |
| **26. Derivation of themes** | **Were themes identiﬁed in advance or derived from the data?** | Themes were derived from the Framework that was used for the analysis |
| **27. Software** | **What software, if applicable, was used to manage the data?** | Data were transcribed verbatim into word document by Sushama Kanan for Bangladesh, Saima Afaq for Pakistan, Anoshmita Adhikary and Vidhya Shree for India and grouped |
| **28. Participant checking** | **Did participants provide feedback on the findings?** | No |
| ***Reporting*** | | |
| **29. Quotations presented** | **Were participant quotations presented to illustrate the themes/ﬁndings? Was each quotation identiﬁed? e.g. participant number** | **Yes** |
| **30. Data and ﬁndings consistent** | **Was there consistency between the data presented and the ﬁndings?** | **Yes** |
| **31. Clarity of major themes** | **Were major themes clearly presented in the ﬁndings?** | **Yes** |
| **32. Clarity of minor themes** | **Is there a description of diverse cases or discussion of minor themes?** | **Yes** |
